# Deep learning based bio-metric authentication system using a high temporal/frequency resolution transform

**DOI:** 10.3389/fdgth.2024.1463713

**Published:** 2024-12-17

**Authors:** Sajjad Maleki Lonbar, Akram Beigi, Nasour Bagheri, Pedro Peris-Lopez, Carmen Camara

**Affiliations:** ^1^ CPS^2^ Lab, Department of Communication, Faculty of Electrical Engineering, Shahid Rajaee Teacher Training University, Tehran, Islamic Republic of Iran; ^2^ Department of AI, Faculty of Computer Engineering, Shahid Rajaee Teacher Training University, Tehran, Islamic Republic of Iran; ^3^ School of Computer Science, Institute for Research in Fundamental Sciences (IPM), Tehran, Iran; ^4^ Computer Science Department, Carlos III University of Madrid, Getafe, Spain

**Keywords:** identity authentication, ECG signal, Wigner-Ville distribution, convolutional neural networks (CNNs), GoogleNet architecture, signal preprocessing, classification deep learning based bio-metric authentication

## Abstract

**Introduction:**

Identity verification plays a crucial role in modern society, with applications spanning from online services to security systems. As the need for robust automatic authentication systems increases, various methodologies—software, hardware, and biometric—have been developed. Among these, biometric modalities have gained significant attention due to their high accuracy and resistance to falsification. This paper focuses on utilizing electrocardiogram (ECG) signals for identity verification, capitalizing on their unique, individualized characteristics.

**Methods:**

In this study, we propose a novel identity verification framework based on ECG signals. Notable datasets, such as the NSRDB and MITDB, are employed to evaluate the performance of the system. These datasets, however, contain inherent noise, which necessitates preprocessing. The proposed framework involves two main steps: (1) signal cleansing to remove noise and (2) transforming the signals into the frequency domain for feature extraction. This is achieved by applying the Wigner-Ville distribution, which converts ECG signals into image data. Each image captures unique cardiac signal information of the individual, ensuring distinction in a noise-free environment. For recognition, deep learning techniques, particularly convolutional neural networks (CNNs), are applied. The GoogleNet architecture is selected for its effectiveness in processing complex image data, and is used for both training and testing the system.

**Results:**

The identity verification model achieved impressive results across two benchmark datasets. For the NSRDB dataset, the model achieved an accuracy of 99.3% and an Equal Error Rate (EER) of 0.8%. Similarly, for the MITDB dataset, the model demonstrated an accuracy of 99.004% and an EER of 0.8%. These results indicate that the proposed framework offers superior performance in comparison to alternative biometric authentication methods.

**Discussion:**

The outcomes of this study highlight the effectiveness of using ECG signals for identity verification, particularly in terms of accuracy and robustness against noise. The proposed framework, leveraging the Wigner-Ville distribution and GoogleNet architecture, demonstrates the potential of deep learning techniques in biometric authentication. The results from the NSRDB and MITDB datasets reflect the high reliability of the model, with exceptionally low error rates. This approach could be extended to other biometric modalities or combined with additional layers of security to enhance its practical applications. Furthermore, future research could explore additional preprocessing techniques or alternative deep learning architectures to further improve the performance of ECG-based identity verification systems.

## 1 Introduction

Security and authentication are among the most important challenges in today’s society. Authentication is the process of accurately verifying the identity of a user, device, or any other entity in a computer system, often as a prerequisite for granting access to system resources (1). Authentication algorithms can generally be classified into three main categories: information-based, token-based, and biometric-based. The biometric-based approach works by matching biometrics such as voice, fingerprint, iris, signature, or DNA features (2). Biometric-based methods operate based on behavioral and biological characteristics. These features are reliable and cannot be forgotten or lost (3). However, there have been instances of face video hacking, fingerprint forgery, and iris forgery in recent years (4). Recently, electrocardiogram (ECG) signals have been used in authentication systems as a reliable biometric-based method (5, 6). ECG biometrics have several advantages compared to other biometric features (2, 5):

• ECG signals are difficult to falsify.
• These signals inherently exist and indicate that the person is alive.
• The ECG signal provides combined information, including both identity information and the health status and condition of the person (1, 4, 7).

Biometric systems that utilize a person’s heart signal have gained popularity due to the simplicity of data acquisition. Biometric authentication, particularly through electrocardiogram (ECG) signals, is emerging as a next-generation technology for both user authentication and physiological health monitoring. The unique liveness detection capability of ECG-based biometric systems distinguishes them from traditional systems, offering enhanced privacy, security, and robustness in authentication (8).

In recent years, growing interest has emerged in investigating the feasibility of using “hidden” biometric traits that include an “aliveness” component, such as electrocardiogram (ECG) or heart signals. These traits offer promising potential for authentication in real-world applications, making ECG signals a more viable option for secure identification (9). Potential applications are extensive and include, but are not limited to, mobile device authentication, access control for restricted areas, data protection on portable devices, banking security, access control for autonomous vehicles and transportation systems, telework verification, continuous authentication and real-time monitoring, as well as identity and status monitoring in emergency and public safety scenarios (10–12).

The main challenges in the literature are (i) the noise components in cardiac signals, (ii) the difficulty of automatically extracting feature sets, and (iii) the performance and efficiency of the system (8). In this research, a biometric-based authentication system that uses the heart rate signal (ECG) is developed. ECG signals are influenced by physiological elements such as emotions, physical activity, and mental activity. ECG signal pre-processing can reduce noise components. One of the most important goals in ECG signal analysis is to obtain a feature space of the signal to fully understand its essence and identify features with high discriminatory power, essential for signal classification. These features are present in both the time and frequency domains. For this purpose, the Wigner-Ville distribution (WVD) has been used in this research (13). Due to the use of the WVD, the signal data is prepared for application in a convolutional neural network. At present, deep neural networks are highly regarded for their efficiency and high detection power in data classification. Therefore, in the present study, we used one of the most significant convolutional neural network architectures, GoogleNet, to develop an ECG-based authentication system. The following issues are covered in this paper:

• We perform appropriate pre-processing on the ECG data to remove signal noise and obtain more accurate findings.
• We define a signal’s Wigner-Ville distribution so that it can be used as an input for a deep neural network. WVD provides a new approach for authenticating ECG data based on convolutional neural networks.
• We propose a highly accurate authentication method for ECG signals using the GoogleNet architecture.

The rest of the paper is organized as follows: In the next section, we review some related work. In Section 3, we discuss the proposed signal pre-processing and the applied convolutional neural network architecture. Section 4 presents the experiments and analysis of the results, and finally, in Section 5, we present the conclusion.

## 2 Related works

In recent years, there has been significant development in ECG signal extraction technology, leading to the growth of biometric-based authentication systems using ECG signals. Numerous research studies have been conducted in this field. The authentication of ECG-based biometric systems can be categorized into fiducial methods, non-fiducial methods, and hybrid methods based on machine learning.

### 2.1 Fiducial methods

Fiducial methods are the earliest approaches used for ECG identification. These methods rely on the morphological characteristics of the ECG signal, such as wavelength, amplitude, peaks, angle or slope of the waveform, and their reference points. Key points such as P, Q, R, S, T, and U are determined to extract the morphological features (14, 15). However, fiducial methods are sensitive to signal processing techniques and heavily rely on heartbeat segmentation and waveform identification algorithms (7). The QRS complex-based features have been widely used for biometric tasks due to their lower sensitivity to physical and emotional changes compared to other parts of the ECG signals. The Pan-Tompkins algorithm (16) is the most commonly used algorithm for detecting different points in the ECG signal, specifically designed for real-time QRS detection (17).

In a study by (18), a mobile biometric authentication system based on electrocardiogram is proposed. This system requires the user to touch the two ECG electrodes of the mobile device for access. However, this study only utilizes one feature, resulting in the relatively poor accuracy (81.82%). Another study (19) states that automatic ECG analysis algorithms extract geometric features and frequency characteristics of ECG signals, greatly facilitating the automatic diagnosis of heart disease. Although this research achieves high accuracy using four features, the applied algorithm is complex and lacks generalizability.

### 2.2 Non-fiducial methods

Non-fiducial methods aim to reduce the complexity of detecting signal points and improve generalization by converting the time domain to frequency domains for feature extraction (7). These methods have lower complexity compared to fiducial methods but heavily rely on signal extraction techniques. The most commonly used signal transformations from time domain to frequency include discrete cosine transform (DCT) (14, 20), Walsh-Hadamard transform (WHT) (21), discrete Fourier transform (DFT) (22), discrete wavelet transform (DWT) (23, 24), and generalized S-transform (GST) (17).

In a study by (20) utilizing the DCT transformation the R-R peak of the signal is first calculated, followed by pre-processing. Then, a DCT transform is applied to each period. For authentication, the correlation between the outputs of the transformed signals should be above 95%. If this correlation is established for three consecutive signals, access is granted; otherwise, access is temporarily restricted. This research reports an accuracy of 97.87% and a processing time of 1.21 s for the authentication of 15 individuals.

Another article (14) uses a band-pass filter to check the signal quality and then extracts features using automatic correlation. Additionally, the Walsh-Hadamard transform is employed for feature transformation, and linear discriminant analysis is used to reduce the dimensionality of the feature vector. The best identification rates reported are 95% for the MIT-BIH arrhythmia database and 97% for the QT database, respectively. In a study by (25), a classifier model is designed to classify ECG signals in the MIT-BIH database. Features are extracted using the S-transform, and a genetic algorithm is utilized to optimize the extracted features in the second step. The final step involves classifying the ECG signals for arrhythmia detection. This study classifies the data into six categories.

### 2.3 Machine learning and hybrid methods

Although fiducial and non-fiducial methods have traditionally been used for ECG signal authentication due to their low complexity, these methods often suffer from errors. In recent years, there has been a growing interest in machine learning-based methods to address these limitations. Some widely used algorithms in these methods include support vector machines (SVM), k-nearest neighbors (KNN), decision trees, random forests, and deep learning algorithms, such as convolutional neural networks (CNNs) and recurrent neural networks (RNNs).

Pinto et al. (26) proposed a method that utilizes feature extraction using Discrete Cosine Transform (DCT) and matching using SVM. Rastogi et al. (27) introduced an authentication system using ECG signals that employs algorithms or techniques such as SVM for authentication purposes and dynamic time warping for signal matching. Agrafioti et al. (28) suggested feature extraction using autocorrelation coefficients (AC) and matching by applying artificial neural networks (ANN). Tan et al. (29) introduced a technique for authentication using mobile sensors, employing the methods of discrete wavelet transform (DWT) and random forest. Sidek et al. (30) suggested the use of KNNs for a biometric system targeting abnormal heart conditions.

In a study (3), effective parts of ECG signals were extracted using empirical mode decomposition (EMD). Feature extraction was performed using statistical, time, and frequency domain features, and the SVM-C method achieved a classification accuracy of 98.72%. The dataset for this study consisted of samples from 14 individuals. In another study (31), a robust pre-processing method was proposed, including noise removal, heart rate normalization, and quality measurement. The ECG signal was decomposed into intrinsic mode functions, and Walsh spectral analysis was used for feature extraction. The KNN method was employed for classification, achieving 95% identification accuracy for 90 individuals. The research (32) focused on determining the minimum number of heartbeats required for authentication. The study used ECG signals from 80 healthy individuals and applied feature extraction using Discrete Wavelet Transform. The random forest algorithm was used for authentication, achieving full accuracy for the dataset.

Recent studies have shown a shift towards deep neural networks, particularly CNNs and RNNs, due to their improved efficiency and accuracy in the field of ECG signal authentication. Salloum et al. (33) proposed an authentication approach based on LSTM networks, a type of RNN (Long Short-Term Memory network), achieving full accuracy. Labati et al. (34) presented the Deep-ECG model, which utilizes deep CNNs to extract key features from one or more leads, resulting in high accuracy. The utilization of CNNs for ECG biometric investigation was first introduced in the study by Kim et al. (4), where a 2D image with three signal periods of ECG signals was used as input to the CNN for authentication.

Further studies on this topic are discussed in references (2, 7, 17, 35), with a comparative analysis provided in (35). Convolutional Neural Networks (CNNs) are widely utilized in these studies, employing various architectures such as VGGNet, ResNet, and GoogleNet. Hong et al. (36) proposed template-free techniques based on deep learning, utilizing the Inception-v3 architecture and achieving an accuracy of 97.84%. Similarly, Zhang et al. (15) developed a human identification system leveraging deep CNNs and Transfer Learning, using multi-view feature representations of ECG signals with the GoogleNet architecture, achieving an accuracy of 97.6%. However, it is important to note that CNNs can be computationally intensive, and the large size of ECG databases poses challenges for real-world applications (37). In a recent study by Aleidan et al., a biometric-based human identification system is presented using an ensemble-based approach and ECG signals. To enhance accuracy and computational efficiency, they employed an ensemble method based on VGG16 pre-trained transfer learning (TL) and Long Short-Term Memory (LSTM) architectures for feature optimization (38). In the study by Agrawal et al., a person authentication system was proposed based on ECG signals using deep learning algorithms. This method comprises two main components: CNNs for feature extraction from ECG data and an LSTM network to model temporal dependencies in the data (39). Likewise, in the work of Parkash et al., a multiplication-based model is proposed, adapting deep learning methods to address limitations of traditional techniques. Their approach includes ECG signal denoising, R-peak detection and segmentation in the preprocessing stage. Cleaned ECG signals are then converted to grayscale images and fed into a customized deep learning model, which includes a novel activation function designed to accelerate network convergence. This model is capable of automatically extracting input data features (8).

In this study, the GoogleNet architecture was chosen for its high speed and accuracy in achieving the desired goal.

## 3 Proposed authentication system

### 3.1 Data

MITDB and NSRDB (40) are two widely used databases for ECG signals, which have been utilized in this research to evaluate the proposed system. The MITDB arrhythmia database comprises 48 individuals, from whom signals were obtained for half an hour each. This collection includes both inpatients (approximately 60%) and outpatients (approximately 40%). Due to the complexity of their signals, this database is a suitable choice for evaluating authentication systems (41). The signals were recorded at a frequency of 360 samples per second and digitized in each channel with an 11-bit resolution within the range of 10 millivolts. It contains ECG signals from 26 men (ranging from 32 to 89 years old) and 22 women (ranging from 23 to 89 years old). The NSRDB dataset consists of 18 long-term ECG recordings from individuals, with a sampling rate of 128 Hz. The individuals in this dataset did not exhibit any significant arrhythmia. It comprises 5 men between the ages of 26 and 45, and 13 women between the ages of 20 and 50.

### 3.2 Preprocessing steps using a high temporal/frequency resolution transform

Preprocessing is essential for reducing noise and extracting meaningful features from the ECG signal, such as the R peak and the QRS complex, which are critical for constructing the feature vector of the signal. Although high-accuracy datasets for ECG signals are available, pre-processing remains necessary due to the presence of various noise types, including baseline fluctuations (below 0.5 Hz), muscle noise from movement (above 40 Hz), and power line noise (60 Hz) (4). These noise sources can introduce false samples that deviate significantly from the original signal, which may lead to errors in subsequent stages, particularly when applying convolutional neural networks. Removing such erroneous samples during pre-processing is therefore crucial. Additionally, some samples might slightly deviate from the original signal due to residual noise; thus, it is necessary to clean the target signal, creating a refined version that closely resembles the original. This refined signal can then be utilized in the neural network model, ultimately enhancing accuracy and reducing error in the authentication process. A widely used algorithm for pre-processing is the Pan-Tompkins algorithm (42), employed in this study as well. For example, in Kim et al.’s work, the Pan-Tompkins algorithm was applied to identify the R peak and segment the continuous ECG signal into individual periods (4). Similarly, Srivastava et al. used the Pan-Tompkins algorithm to remove noise, segment heartbeats, and convert the ECG signal into two-dimensional images; after detecting the R peaks, the QRS complex was identified, and three consecutive heartbeats were used to extract features with potential variations (43).

One popular algorithm used for pre-processing is the Pan-Tompkins algorithm (42), which is also employed in this research. In Kim et al.’s study, the R-peak was identified based on the Pan-Tompkins algorithm in order to divide the continuous ECG signal into single period parts and the signal was segmented at the detected R-peak (4). Additionally, in Srivastva et al.’s study, the Pan-Tompkins algorithm was used for ECG preprocessing, and its steps include noise removal, heartbeat segmentation, and two-dimensional image conversion of the signal, and after the R peaks of the filtered signal, the QRS is determined. and finally three consecutive heartbeats are used to collect features with possible changes (43).

**Figure 1:**
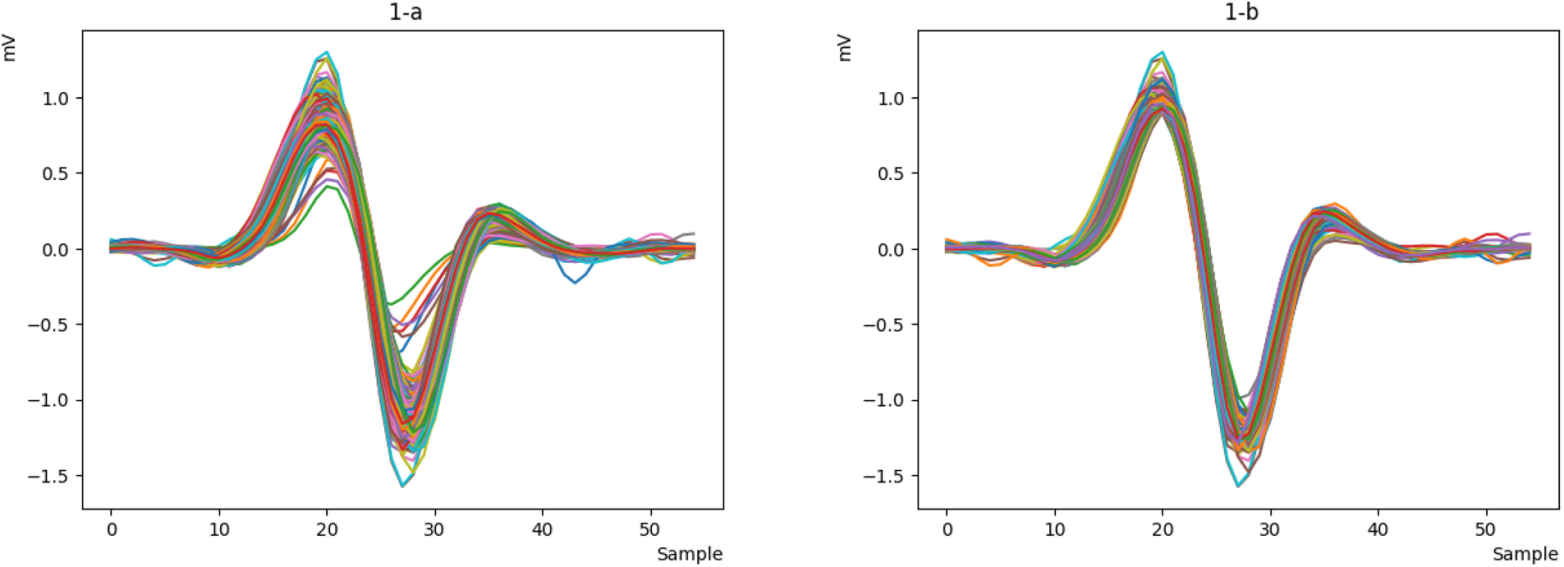
The first signal and the processed signal: (**a**) the first signal. (**b**) processed signal.

The raw ECG signals from the database are processed using the Pan-Tompkins algorithm to identify the P, Q, R, S, and T peaks. The signals are then cleaned and resampled at an appropriate sample rate to reduce noise and generate a smoother signal. Next, the ECG signals are divided into windows, each containing a heartbeat. The R-peak in each window is determined, and the height of the R-peak is measured. The mode of the R-peak heights is calculated. Finally, the signals that are close to this mode are selected as the final signal. Figure 1a depicts the raw data, while Figure 1b shows the processed signal, revealing that a portion of the heart rate has been removed.

In this research, the Wigner-Ville Distribution (WVD) (13) was utilized to enhance the ECG data signal and improve the detection capabilities of the data (44). To complete the pre-processing steps, it was observed that the results of the Wigner-Ville distribution varied depending on each individual’s heart rate. Therefore, the WVD was applied separately to each heartbeat, enabling the conversion of one-dimensional data into two-dimensional images. WVD is a powerful tool in time-frequency analysis, non-stationary signal processing, and some real-world scenarios (45). Also, WVD plays an important role in time-frequency representation because it can provide good energy distribution and high resolution for non-stationary signal processing (46). The following is a detailed description of the methodology and steps involved in this process:

(1) $$W(t,f)=\int f\left(t+\frac{\tau }{2}\right)f^{⋆}\left(t-\frac{\tau }{2}\right)\;e^{-j2\pi f\tau }\;d\tau ,\;W(t,f)\in R,\;\forall t,f$$

In Equation 1, the function f(t) is given by the Equation 2:

(2) $$f(t)=X(t)+j\hat{X}(t)$$

In this context, the operator $\hat{X}(t)$ is the Hilbert transform, defined by Equation 3:

(3) $$\hat{X}(t)=X(t)⋆\frac{1}{\pi t}$$

Equation 4 yields the quantity of the signal energy X(t) of a WVD:

(4) E=∫∫W(t,f)dtdf

The spectral energy density and instantaneous power can be obtained from the marginal distribution of W, as it is described by Equation 5 and Equation 6:

(5) $$\int W(t,f)\;df=|X(t){|}^{2}$$

(6) $$\int W(t,f)\;dt=|X(f){|}^{2}$$

The properties of time and frequency shifting in the WVD are formulated in Equations 7, 8:

(7) $$X(t)=Y(t-t_{0})⟺W_{X}(t,f)=W_{Y}(t-t_{0},f)$$

(8) $$X(t)=Y(t)\;e^{-j2\pi f_{0}t}⟺W_{X}(t,f)=W_{Y}(t,f-f_{0})$$

Also, the properties of time and frequency scaling in the WVD are formulated in Equations 9, 10:

(9) $$X(t)=Y(t-t_{0})⟺W_{X}(t,f)=W_{Y}(t-t_{0},f)$$

(10) $$X(t)=\sqrt{k}Y(kt)⟺W_{X}(t,f)=W_{Y}\left(kt,\frac{\;f}{k}\right)$$

Instantaneous frequency is a term that describes the behavior of local frequencies as a function of time (13). Let

(11) $$X(t)=A(t)\;e^{\;j\varphi (t)}$$

Where both the magnitude A(t) and the phase φ(t) are real-valued functions. Then, given Equations 9, 10 and 11, < *f* >_t_ can be determined using Equation 12:

(12) $$<f{>}_{t}=\frac{2\pi \int fW(t,f)\;df}{\int W(t,f)\;df}=\frac{2\pi \int fW(t,f)\;df}{|A(t){|}^{2}}=\varphi^{\prime }(t)$$

In WVD, the local time behavior is defined as a function of frequency via group delay. Suppose the Fourier transform of the signal X(t) is equal to $X(f)=A(f)\;e^{\;j\psi (f)}$. The value of $\psi^{\prime }(t)$ is called the group delay, which is obtained from the Equation 13 for the Wigner-ville distribution (13):

(13) $$\frac{\int tW(t,f)\;dt}{\int W(t,f)\;dt}=\frac{\int tW(t,f)\;dt}{|A(f){|}^{2}}=-\psi^{\prime }(t)$$

**Figure 2:**
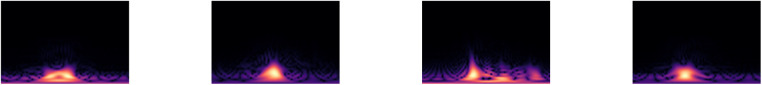
Two-dimensional image of a heartbeat (from different individuals).

We can now extract the WVD of the ECG signal as a two-dimensional image and use it as input for a deep neural network. One crucial aspect of this image is its uniqueness for each sample, which plays a vital role in data authentication. In Figure 2, we present a WVD representation of heart rates from multiple samples. These images are used for training, testing, and evaluation of the deep neural network.

### 3.3 Deep learning based authentication model

**Figure 3:**
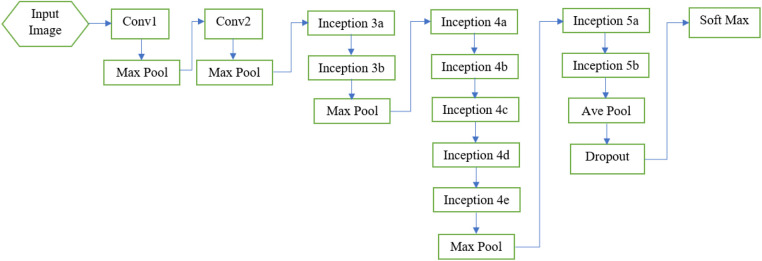
Overview of Google Net layers (37).

Convolutional neural networks (CNNs) are powerful tools for image classification and processing. These networks come in different architectures, each with varying speed, accuracy, and complexity. It has been observed in the literature that increasing the number of layers in a CNN significantly improves its performance. However, increasing the number of layers can be expensive for large networks due to the common problem of over-fitting. To address the challenges faced by large networks, the GoogleNet architecture has been developed, which incorporates the Inception module (37). The GoogleNet architecture has proven effective in solving many of the issues encountered by large networks (47). In this research, we have utilized the GoogleNet architecture to process the image data obtained from the pre-processing step. In the following, we will delve into the details of this network. A diagram of GoogleNet architecture is depicted in Figure 3. Also Table 1 specifies the layers of GoogleNet (37).

**Table 1:** Details of Google Net layers (37).

Type	Patch size/ stride	Output size	Depth	1 × 1	3 × 3 reduce	3 × 3	5 × 5 reduce	5 × 5	Pool proj	Params	ops
Conv	7 × 7/2	112 × 112 × 64	1	–	–	–	–	–	–	2.7K	34M
Max pool	3 × 3/2	56 × 56 × 64	0	–	–	–	–	–	–	–	–
conv	3 × 3/1	56 × 56 × 192	2	–	64	192	–	–	–	112K	360M
Max pool	3 × 3/2	28 × 28 × 192	0	–	–	–	–	–	–	–	–
Inception 3a	–	28 × 28 × 256	2	64	96	128	16	32	32	159K	128M
Inception 3b	–	28 × 28 × 480	2	128	128	192	32	96	64	390K	304M
Max pool	3 × 3/2	14 × 14 × 480	0	–	–	–	–	–	–	–	–
Inception 4a	–	14 × 14 × 512	2	192	96	208	16	48	64	364K	73M
Inception 4b	–	14 × 14 × 512	2	160	112	224	24	64	64	437K	88M
Inception 4c	–	14 × 14 × 512	2	128	128	256	24	64	64	463K	100M
Inception 4d	–	14 × 14 × 528	2	112	144	288	32	64	64	580K	119M
Inception 4e	–	14 × 14 × 832	2	256	160	320	32	128	128	840K	170M
Max pool	3 × 3/2	7 × 7 × 832	0	–	–	–	–	–	–	–	–
Inception 5a	–	7 × 7 × 832	2	256	160	320	32	128	128	1072K	54M
Inception 5b	–	7 × 7 × 1024	2	384	192	384	48	128	128	1388K	71M
Avg pool	7 × 7/1	1 × 1 × 1024	0	–	–	–	–	–	–	–	–
Dropout 40%	–	1 × 1 × 1024	0	–	–	–	–	–	–	–	–
Linear	–	1 × 1 × 1000	1	–	–	–	–	–	–	1000K	1M
Softmax	–	1 × 1 × 1000	0	–	–	–	–	–	–	–	–

In the following, the layers of the GoogleNet architecture will be described.

#### 3.3.1 Convolution layer

The initial layer of the GoogleNet architecture is a convolutional layer that utilizes a (7×7) size filter, as shown in Figure 3. Its main goal is to reduce the input image size without losing spatial information. In this layer, the input image is reduced to a size of (112×112).

#### 3.3.2 Max pooling layer

After the first convolutional layer, there is a max pooling layer that further reduces the image size to (56×56). Before reaching the first Inception layer, there is another max pooling layer that reduces the image size to ($\frac{1}{8}$) of the original. The number of feature maps increases as the image size decreases, as indicated in Table 1, from 64 in the first convolutional layer to 192 in the second convolutional layer (37).

#### 3.3.3 Inception module

The Inception module is a neural network design that utilizes convolutional layers and other filters to perform feature recognition at various sizes, while reducing the computational cost of training a large network by decreasing its dimensionality. The main idea behind the Inception module is to approximate an optimal local sparse structure in a convolutional network. It involves analyzing the correlation statistics of the last layer and clustering them into groups of highly correlated units. These clusters form the units of the next layer, which are connected to the units of the previous layer. Each unit from the previous layer corresponds to a region of the input image and is grouped into filter banks. One of the key advantages of this architecture is that it allows for a significant increase in the number of units in each phase without increasing computational complexity.

**Figure 4:**
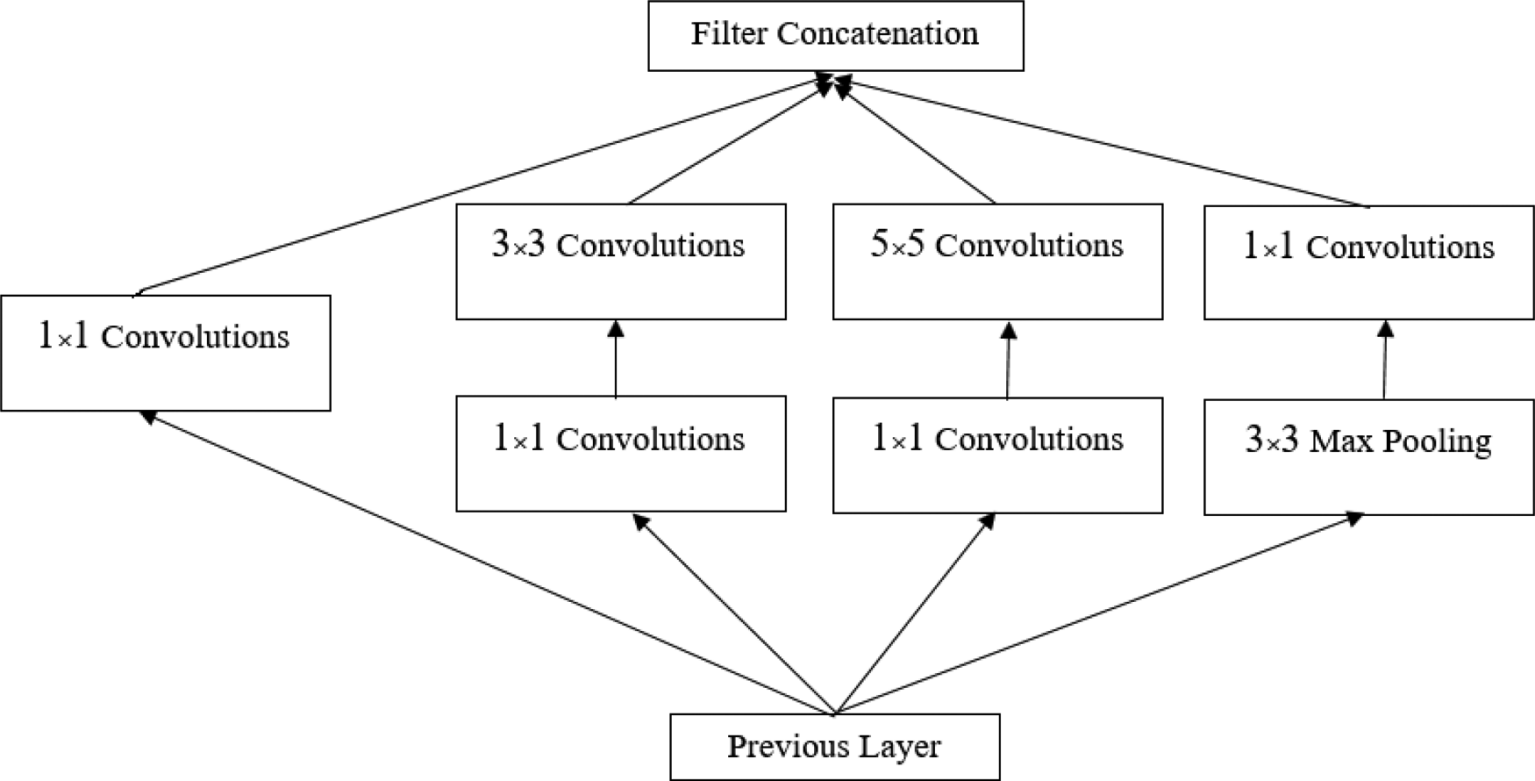
Inception module (37).

After the two convolutional layers, the GoogleNet architecture incorporates nine Inception modules (37), as illustrated in Figure 3. The purpose of these modules is to perform multiple operations in parallel, including integration and convolution, using filters of various sizes. The use of filters with different sizes helps capture diverse features in the images. It is worth mentioning that there are two levels of max pooling between the Inception modules. These max pooling layers are used to further reduce the size of the input as it propagates through the network, effectively reducing the width and height of the image. Additionally, the input image is progressively shrunk within the Inception modules, contributing to a reduction in the network’s computational burden. The architecture of the Inception module is depicted in Figure 4 (37).

#### 3.3.4 Dropout layer

Following this, a dropout layer with a rate of 40% is applied before the fully connected (linear) layer. The dropout layer is a regularization technique used during network training to mitigate overfitting. It helps prevent the network from relying too heavily on specific features by randomly dropping a fraction of the nodes during each training iteration.

#### 3.3.5 Linear layer

The linear layer consists of 1,000 nodes, which correspond to the 1,000 classifications in the ImageNet dataset. This layer performs a linear transformation on the input data, mapping it to a 1,000-dimensional feature space.

#### 3.3.6 Softmax layer

The last layer of the network is a Softmax layer. The Softmax function, an activation function, is used in this layer to calculate the probability distribution of a collection of integers based on an input vector. The Softmax activation function is defined using Equation 14 (37):

(14) $$\sigma (z_{i})=\frac{e^{z_{i}}}{\sum_{\;j=1}^{k}\;e^{z_{\;j}}}\;\mathrm{for}\;i=1,2,\ldots ,k\;and\;\;\;\;z=(z_{1},\ldots ,z_{k})\in R^{k}$$

The Softmax function takes an input vector (z) and computes a vector of values representing the likelihood of each class or event. The sum of all these values equals one, ensuring a valid probability distribution. So far, we have discussed the different layers of the GoogleNet architecture. However, there is another important component of this architecture that we need to address: the Auxiliary Classifier.

#### 3.3.7 Auxiliary classifier

As mentioned earlier, the Vanishing Gradient problem is one of the most significant challenges in neural networks. This problem occurs when the weight updates during backpropagation in the earlier layers become very small, resulting in a diminished gradient. In other words, the network’s training progress slows down or even stops. To mitigate this issue, auxiliary classifiers are added to the middle layers of the architecture, specifically in the eighth (Inception 4a) and eleventh [Inception 4 (d)] layers.

#### 3.3.8 Auxilary classifier function

**Figure 5:**
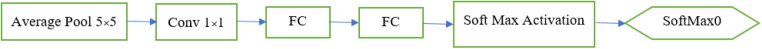
Auxiliary classifier (37).

During training, additional classifications are introduced to the network through these auxiliary classifiers. These classifiers evaluate the data and provide feedback to the middle layers of the network, contributing to the overall loss estimation. The error from the auxiliary classifier is weighted at 0.3, meaning it has a smaller impact on the overall loss compared to the main classifier. An illustration of the auxiliary classifier can be seen in Figure 5 (37).

One of the notable advantages of this architecture is its ability to significantly increase the number of units per stage without a proportional increase in computational complexity. The GoogleNet network is designed with computational and practical efficiency in mind, making it suitable for deployment on devices with limited computational resources and low memory requirements.

## 4 Experiments

In this section, we will describe the simulation method, the tests performed, and the results using real data. As mentioned, we used two datasets: MITDB and NSRDB. It should be noted that all simulations were programmed in Python, and due to the large volume of data and the need for intensive data processing, they were implemented in the Google Colab Pro environment. Google Colab Pro is highly suitable for research involving deep learning, offering 32 GB of RAM with GPU support (P100 and T4).

### 4.1 ECG signal preprocessing

**Figure 6:**
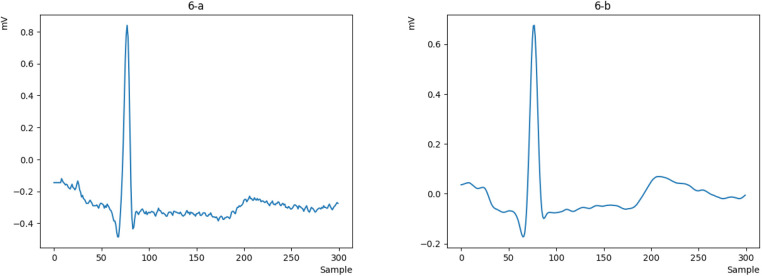
**(a)** The raw ECG signal and **(b)** the cleaned one with Pan Tompkins.

First, we obtain the raw ECG signal from the MITDB and NSRDB datasets using the WFDB library in Python and plot a heartbeat, as shown in Figure 6a. Then, we clean the raw signal using the Pan-Tompkins algorithm to obtain a smooth and continuous signal. The output of the cleaned signal is shown in Figure 6b.

**Figure 7:**
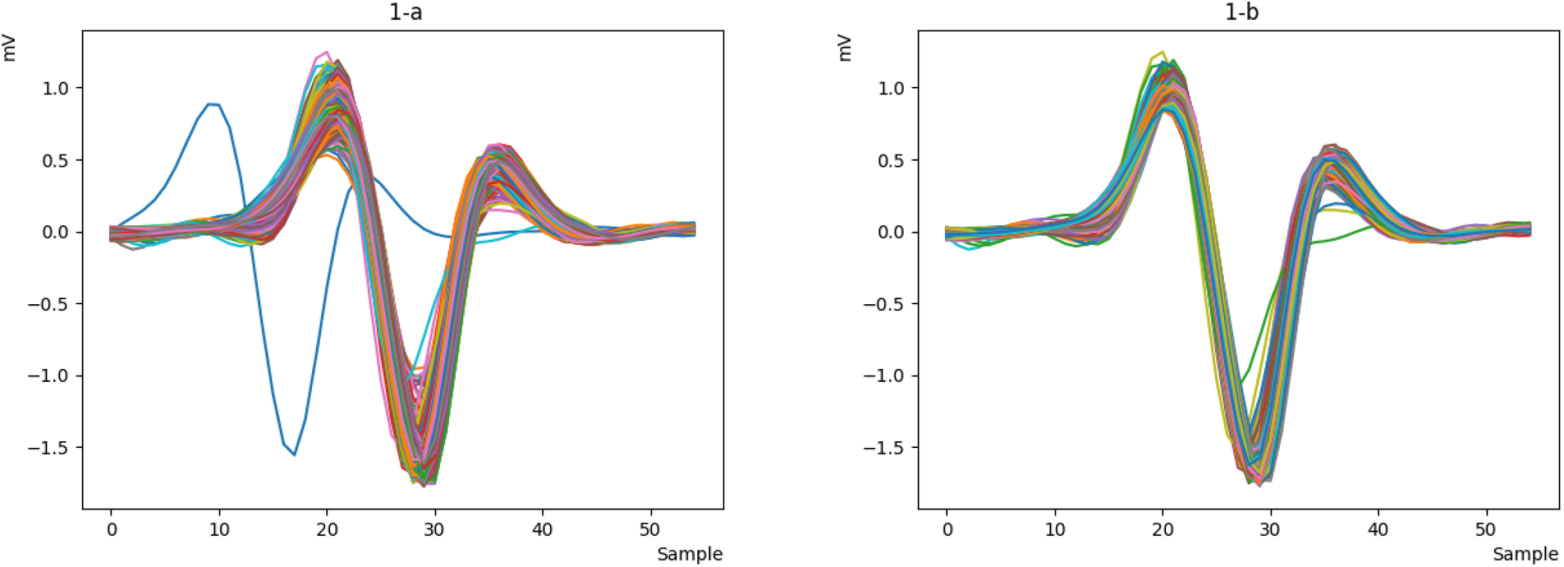
Integrated representation of the heartbeats of sample 122 from the MITDB database (**a**) display all heartbeats. (**b**) remove different heartbeats.

Now that the signal is smooth and clean, we can easily detect the R peak and extract the key characteristics of the signal. However, during signal extraction, noise (such as patient movement or device interference) can cause anomalies, resulting in signals that are inappropriate and need to be removed. This ensures that the neural network is not confused in the future and can more easily recognize valid signals. To better illustrate this issue, we will display all the heartbeats in a single figure for clearer comparison. We will then remove the incorrect signals, as they do not represent valid data from the person and could disrupt the authentication process. As shown in Figure 7a, some signals do not match the original signal, so we remove them to retain only the correct signals, as depicted in Figure 7b.

To remove unbalanced signals, the following steps are applied to the signal:

1. The distance between all R peaks (R-to-R interval) is measured, and its mode is obtained. Then, all signals that deviate by more than 30 samples from the mode are considered incorrect.
2. The size of all R peaks is measured, and their mode is calculated. Any signals with R peak sizes smaller or larger than 0.15 millivolts from the mode are considered incorrect.

In this step of the pre-processing method, the prepared data is then applied to the Wigner-Ville distribution.

### 4.2 Application of Wigner-Ville distribution on the ECG signal

**Figure 8:**
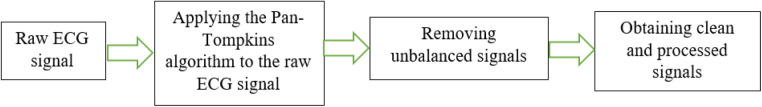
ECG signal preprocessing diagram.

The proposed preprocessing steps are shown in Figure 8. As mentioned before, they are as follows:

• Applying Pan-Tompkins algorithm to the raw ECG signals and separating the ECG signals into unit heartbeats.
• Applying WVD to each person’s heartbeat separately, as they are different.
• Transforming 1D vector to 2D image data.

In the study by Akdeniz et al., an ECG arrhythmia detection algorithm based on the Wigner-Ville Distribution is proposed, which monitors chronic patients through a telemedicine system utilizing contemporary mobile information and communication technologies (48). Similarly, in the study by Desai et al., electrocardiogram (ECG) signals were employed to identify various cardiovascular diseases and abnormalities. In this research, ECG signals were converted into time-frequency images using the pseudo-Wigner-Ville Distribution. These images are divided into training and testing datasets and subsequently fed into a Convolutional Neural Network (CNN) model to differentiate between normal and abnormal heartbeats. This method demonstrates significant clinical applications (49).

The primary rationale for using this distribution is the need for input in the form of images, and we identified it as a suitable input format for the two-dimensional convolutional neural network. To visualize the Wigner-Ville Distribution, we utilized the wigner_ville_spectrum library in Python, with the following settings:

• wigner_ville_spectrum(data, delta, time_bandwidth, smoothing_filter)
• The data parameter is the same as the input signal, one heartbeat from each sample is considered, and its type is numpy.ndarray.
• The delta parameter is the data sampling interval, which we have considered as 10.
• The time_bandwidth parameter is the bandwidth time, which is considered equal to 3.5.
• Finally, we have set the parameter type of smoothing_filter as Gaussian. The above values are considered experimentally.

### 4.3 Experimental setup and evaluation metrics

In this work, the performance of the proposed ECG identification method is evaluated on the MITDB and NSRDB datasets. In this model, the ratio of training, evaluation, and test datasets is 80, 10, and 10 respectively. The following are the necessary parameters used to define the metrics:

**True positive (TP):** A test result that correctly shows the existence of a condition or characteristic.

**False positive (FP):** A test result that falsely indicates that a certain condition or characteristic is present.

**False negative (FN):** A test result that falsely indicates that a certain condition or characteristic is not present.

**True negative (TN):** A test result that correctly indicates the absence of a condition or characteristic.

The performance of the proposed biometric system is evaluated using the following metrics:

**Accuracy:** In a system, accuracy is equal to the ratio of the number of correct predictions to the total number of predictions made. If we consider the correct predictions TP and TN, the accuracy is calculated from Equation 15.

(15) $$Accuracy=\frac{TN+TP}{TN+TP+FN+FP}$$

**Precision:** According to Equation 15, accuracy alone does not distinguish between False Negatives (FN) and False Positives (FP). To differentiate them, the Precision metric is defined as follows:

(16) $$Precision=\frac{TP}{TP+FP}$$

Based on Equation 16, we can see that the Precision metric focuses on the model’s positive predictions and determines what percentage of those positive predictions were correct.

**Recall:** This metric was introduced to address the limitations of Precision. It focuses on the data that is truly positive and is calculated from Equation 17. The Recall metric is sometimes referred to as the sensitivity metric.

(17) $$Recall=\frac{TP}{TP+FN}$$

**F1 score:** This metric is a combination of precision and recall, and for unbalanced data, it can serve as a good benchmark for comparison. It is obtained from Equation 18.

(18) $$F1=\frac{2(Precision\times Recall)}{Precision+Recall}$$

**False Acceptance Rate (FAR):** It is defined as the ratio of the number of false acceptances to the total number of authentication attempts (Equation 19):

(19) $$FAR=\frac{FN}{TP+FN}$$

**False Rejection Rate (FRR):** It is defined as the ratio of the number of false rejections to the total number of authentication attempts (Equation 20):

(20) $$FRR=\frac{FP}{FN+FP}$$

**Equal Error Rate (EER):** The error rate at which FAR and FRR are equal (the point of compromise between FAR and FRR).

### 4.4 Results for database MITDB and NSRDB

**Figure 9:**
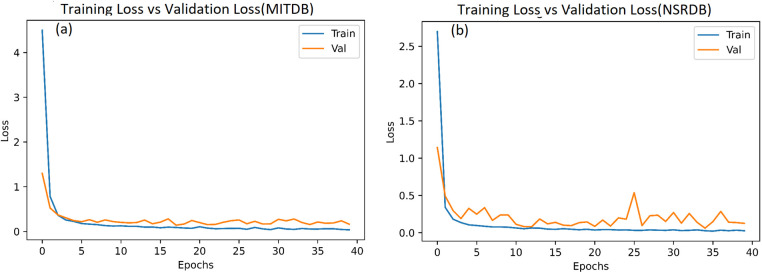
Loss function after 40 epochs: **(a)** MITDB database. **(b)** NSRDB database.

**Figure 10:**
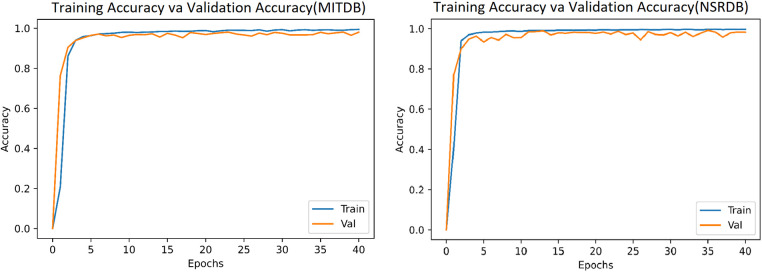
Model recognition accuracy after 40 epochs: **(a)** MITDB database. **(b)** NSRDB database.

In this system, we have used 34,713 and 36,000 images (heartbeats converted into images) from the MITDB and NSRDB databases, respectively. The model is simulated with Epoch = 40 and batch_size = 64, and the results after 40 epochs for the MITDB and NSRDB databases are shown in Figures 9, 10. We achieve very high accuracy in the initial steps. Additionally, the accuracy of the model surpasses 90% after just 3 epochs, making this approach suitable for applications where the speed of training is of great importance.

Table 2 shows a comparison between the results of running the model on the MITDB and NSRDB datasets. The model requires 1.4 and 1.3 ms per heartbeat for authentication, respectively.

**Table 2:** Comparison of MITDB and NSRDB results.

Parameter	NSRDB	MITDB
Total number of images	36,000	34,713
Total number of images	34,713	36,000
RAM is occupied	22	24
Training duration (For a heartbeat)	73 ms	71 ms
Authentication duration (For a heartbeat)	1.4 ms	1.3 ms
Precision	99.1	98.65
Recall	99.3	98.67
F1	99.2	98.66
Loss	0.028	0.065
Accuracy	99.3	99.004
FAR	0.7	1.33
FRR	0.9	1.35
EER	0.8	1.34

**Figure 11:**
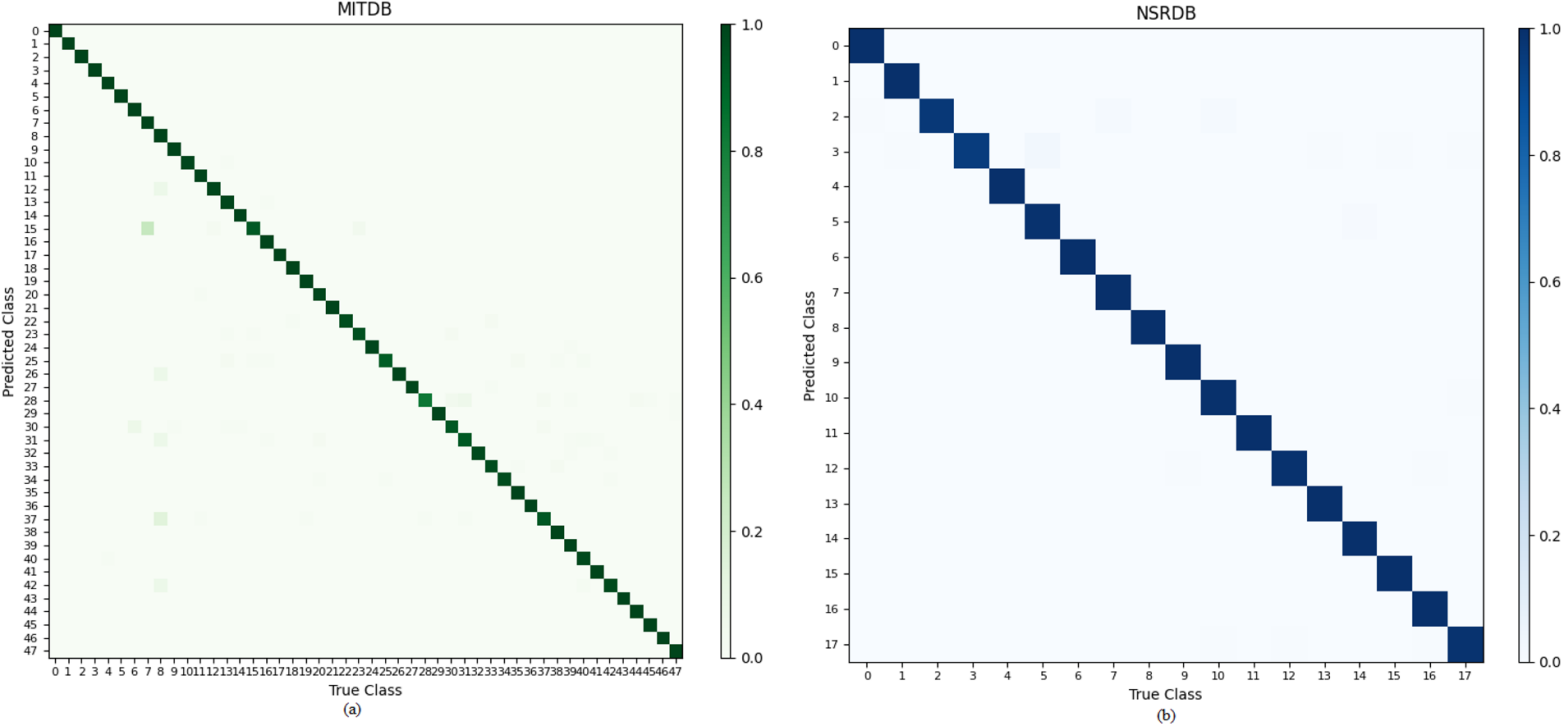
Confusion matrix for the classes of the two databases: **(a)** MITDB database. **(b)** NSRDB database.

The confusion matrix for the classes of the two databases is shown in Figure 11.

### 4.5 Comparative analysis

Based on the obtained results, we conducted a comprehensive analysis and comparison of previous studies in the field of ECG authentication using the MITDB and NSRDB databases, alongside our own study. As we know, each database has its own characteristics. For instance, previous research has indicated that achieving high accuracy in the MITDB database is more challenging compared to other databases. While some studies have achieved 100% accuracy in certain databases, such a result has not yet been reported in the MITDB database.

One of the strengths of our system is that it allows for a comprehensive comparison of the proposed method with recent research, not limited to neural network-based approaches. In fact, we have conducted this comparison by considering the latest published studies on the MITDB and NSRDB databases. See Tables 3, 4 for the results on the MITDB and NSRDB, respectively.

**Table 3:** Comparison of authentication model accuracy on the NSRDB dataset.

Authors	Method	Accuracy	EER
Zhang et al. (50)	CWT,CNN	95.1	–
Mai et al. (51)	QRS,MLP	98	2
Kim et al. (4)	CNN	98.2	–
Tan et al. (29)	DWT	99	–
Proposed	CNN	99.3	0.8

**Table 4:** Comparison of authentication model accuracy on the MITDB dataset.

Authors	Method	Accuracy	EER
Zhang et al. (50)	CWT,CNN	91.1	–
Wang et al. (55)	MSDF	94.68	5.3
Chu et al. (7)	CNN	95.99	4.74
Salloum et al. (33)	LSTM	96	3.5
Wang et al. (56)	FV,CNN	97.66	–
Fatimah et al. (57)	FD,ML	97.92	–
Belo et al. (54)	RNN	97.92	–
Tan et al. (29)	DWT	98	–
Li et al. (53)	GNMF	98.03	10.44
El Boujnouni et al. (58)	WT,CN	98.2	–
Lynn et al. (52)	CNN	98.4	–
Meltzer et al. (59)	AC,DCT	98.8	–
Proposed	CNN	99.004	1.34

According to Tables 3, 4, the proposed model in this study achieved accuracies of 99.004% and 99.3% for the MITDB and NSRDB databases, respectively. These accuracies are the highest reported among studies conducted on these two databases.

The proposed system processes a single heartbeat signal for authentication, whereas the study (52) used 3 and 9 heartbeats. Additionally, that study utilized a combination of LSTM and CNN methods to achieve the reported results, which increases the complexity. In the study (53), 3 heartbeats were used for authentication, and the authentication duration was 7 ms. In contrast, our proposed system requires only 1.3 ms for authentication.

The study (54) employed the RNN method. One advantage of this system, compared to our proposed approach, is that all models work independently, and there is no need to retrain all the models when adding a new person. However, despite being less accurate than our proposed system, this system has significantly higher complexity, resulting in a lengthy training time for each person. In the aforementioned study, using the Nvidia GTX 1080Ti graphics processor, the average training time was 36 h, which is impractical for real-world applications. Also, according to recent published reports, Table 3 compares the FAR metric, and Table 4 compares the EER metric.

## 5 Conclusion

This research focuses on the authentication of ECG signals and utilizes two databases, MITDB and NSRDB, for analysis. The signals were processed using the Pan-Tompkins algorithm, followed by the removal of segments that deviated significantly from expected patterns to extract a clean representation. The extraction of a clean signal is critical, as it directly impacts the accuracy of the study. In this research, signals from individuals with minimal noise and effective cleaning achieved a 100% accuracy rate. For example, the recall metric for the NSRDB database achieved a 100% success rate for 12 individuals, while in the MITDB database, the recall metric also reached 100% for 31 individuals. These results underscore the potential for perfect accuracy with meticulous signal acquisition. Each processed signal was then transformed into the frequency domain, where the Wigner-Ville distribution was applied to generate images that served as inputs for the deep neural network. These frequency-domain images were subsequently input into the GoogleNet architecture, leading to the development of an authentication system. This model achieved accuracies of 99.004% and 99.3% for the MITDB and NSRDB databases, respectively. These accuracy rates surpass those of other studies while maintaining a lower level of complexity. Overall, this research demonstrates the significance of signal cleaning, frequency domain analysis, and the utilization of convolutional neural networks and deep learning in achieving high accuracy and reduced complexity in ECG signal authentication.

## Data Availability

The original contributions presented in the study are included in the article/Supplementary Material, further inquiries can be directed to the corresponding author.

## References

[1] Agrafioti F Hatzinakos D. ECG biometric analysis in cardiac irregularity conditions. In: Signal, Image and Video Processing (SIViP). Volume 3. Florham Park, NJ: Springer (2009). p. 329–43.

[2] Hammad M Liu Y Wang K. Multimodal biometric authentication systems using convolution neural network based on different level fusion of ECG and fingerprint. IEEE Access. (2019) 7:26527–42. 10.1109/ACCESS.2018.2886573

[3] Aziz S Khan MU Choudhry ZA Aymin A Usman A. ECG-based biometric authentication using empirical mode decomposition and support vector machines. In: *2019 IEEE 10th Annual Information Technology, Electronics and Mobile Communication Conference (IEMCON)*. IEEE (2019). p. 0906–12.

[4] Kim JS Kim SH Pan SB. Personal recognition using convolutional neural network with ECG coupling image. J Ambient Intell Humaniz Comput. (2020) 11(5):1923–32. 10.1007/s12652-019-01401-3

[5] Huang Y Yang G Wang K Liu H Yin Y. Learning joint and specific patterns: a unified sparse representation for off-the-person ECG biometric recognition. IEEE Trans Inf Forensics Secur. (2021) 16:147–60. 10.1109/TIFS.2020.3006384

[6] Lonbar SM Beigi A Bagheri N. Electrocardiogram signal authentication system based on deep learning. Biannual J Monadi Cyberspace Secur. (2024) 12(2):33–41. https://monadi.isc.org.ir/browse.php?a_id=251&sid=1&slc_lang=en

[7] Chu Y Shen H Huang K. ECG authentication method based on parallel multi-scale one-dimensional residual network with center and margin loss. IEEE Access. (2019) 7:51598–607. 10.1109/ACCESS.2019.2912519

[8] Prakash AJ Patro KK Samantray S Plawiak P Hammad M. A deep learning technique for biometric authentication using ECG beat template matching. Information. (2023) 14(2):65. 10.3390/info14020065

[9] Yuniarti AR Rizal S Lim KM. Single heartbeat ECG authentication: a 1D-cnn framework for robust and efficient human identification. Front Bioeng Biotechnol. (2024) 12:1398888. 10.3389/fbioe.2024.1398888 39027407 PMC11254790

[10] Bhuva DR Kumar S. A novel continuous authentication method using biometrics for IOT devices. Internet Things. (2023) 24:100927. 10.1016/j.iot.2023.100927

[11] Camara C Peris-Lopez P González-Manzano L Tapiador JE. Real-time electrocardiogram streams for continuous authentication. Appl Soft Comput. (2018) 68:784–94. 10.1016/j.asoc.2017.07.032

[12] Uwaechia AN Ramli DA. A comprehensive survey on ECG signals as new biometric modality for human authentication: recent advances and future challenges. IEEE Access. (2021) 9:97760–802. 10.1109/ACCESS.2021.3095248

[13] Quian S Chen D. Joint Time-Frequency Analysis, Methods and Applications. New York: Prentince-Hall PTR (1996).

[14] Srivastva R Singh YN. Human recognition using discrete cosine transform and discriminant analysis of ECG. In: *2017 Fourth International Conference on Image Information Processing (ICIIP)*. IEEE (2017). p. 1–5.

[15] Zhang Y Zhao Z Deng Y Zhang X Zhang Y. Human identification driven by deep CNN and transfer learning based on multiview feature representations of ECG. Biomed Signal Process Control. (2021) 68:102689. 10.1016/j.bspc.2021.102689

[16] Pan J Tompkins WJ. A real-time qrs detection algorithm. IEEE Trans Biomed Eng. (1985) BME-32(3):230–6. 10.1109/TBME.1985.325532 3997178

[17] Zhao Z Zhang Y Deng Y Zhang X. ECG authentication system design incorporating a convolutional neural network and generalized s-transformation. Comput Biol Med. (2018) 102:168–79. 10.1016/j.compbiomed.2018.09.027 30290297

[18] Arteaga-Falconi JS Al Osman H El Saddik A. Ecg authentication for mobile devices. IEEE Trans Instrum Meas. (2015) 65(3):591–600. 10.1109/TIM.2015.2503863

[19] Liu B Liu J Wang G Huang K Li F Zheng Y, et al. A novel electrocardiogram parameterization algorithm and its application in myocardial infarction detection. Comput Biol Med. (2015) 61:178–84. 10.1016/j.compbiomed.2014.08.010 25201457

[20] Hussein AF AlZubaidi AK Al-Bayaty A Habash QA. An iot real-time biometric authentication system based on ECG fiducial extracted features using discrete cosine transform. *CoRR* [Preprint]. *abs/1708.08189* (2017).

[21] Srivastva R Singh YN. ECG biometric analysis using walsh–hadamard transform. In: *Advances in Data and Information Sciences: Proceedings of ICDIS-2017*. Springer (2018). Vol. 1. p. 201–10.

[22] Srivastva R Singh YN. Identifying individuals using fourier and discriminant analysis of electrocardiogram. In: Ghosh D, Giri D, Mohapatra RN, Savas E, Sakurai K, Singh LP, editors. *Mathematics and Computing – 4th International Conference, ICMC 2018, Varanasi, India, January 9–11, 2018, Revised Selected Papers*. Communications in Computer and Information Science. Springer (2018). Vol. 834. p. 286–95.

[23] Chiu C-C Chuang C-M Hsu C-Y. A novel personal identity verification approach using a discrete wavelet transform of the ECG signal. In: *2008 International Conference on Multimedia and Ubiquitous Engineering (MUE 2008), 24–26 April 2008, Busan, Korea*. IEEE Computer Society (2008). p. 201–6.

[24] Kaur I Rajni R Marwaha A. Ecg signal analysis and arrhythmia detection using wavelet transform. J Inst Eng Ser B. (2016) 97:499–507. 10.1007/s40031-016-0247-3

[25] Das MK Ghosh DK Ari S. Electrocardiogram (ECG) signal classification using s-transform, genetic algorithm and neural network. In: *2013 IEEE 1st International Conference on Condition Assessment Techniques in Electrical Systems (CATCON)*. IEEE (2013). p. 353–7.

[26] Pinto JR Cardoso JS Lourenço A Carreiras C. Towards a continuous biometric system based on ECG signals acquired on the steering wheel. Sensors. (2017) 17(10):2228. 10.3390/s17102228 28956856 PMC5676989

[27] Rastogi R Mittal A Verma I Saxena P. Using ECG authentication for biometrics in smart cities. Int J Syst Software Secur Prot. (2023) 14(1):1–26. 10.4018/IJSSSP.324078

[28] Agrafioti F Hatzinakos D. ECG biometric analysis in cardiac irregularity conditions. Signal Image Video Process. (2009) 3(4):329–43. 10.1007/s11760-008-0073-4

[29] Tan R Perkowski MA. Toward improving electrocardiogram (ECG) biometric verification using mobile sensors: a two-stage classifier approach. Sensors. (2017) 17(2):410. 10.3390/s17020410 28230745 PMC5335986

[30] Sidek KA Khalil I Jelinek HF. ECG biometric with abnormal cardiac conditions in remote monitoring system. IEEE Trans Syst Man Cybern Syst. (2014) 44(11):1498–509. 10.1109/TSMC.2014.2336842

[31] Zhao Z Yang L Chen D Luo Y. A human ECG identification system based on ensemble empirical mode decomposition. Sensors. (2013) 13(5):6832–64. 10.3390/s130506832 23698274 PMC3690084

[32] Belgacem N Nait-Ali A Fournier R Bereksi-Reguig F. Ecg based human authentication using wavelets and random forests. Int J Cryptogr Inf Secur. (2012) 2(2):1–11. 10.5121/IJCIS.2012.2201

[33] Salloum R Jay Kuo C-C. ECG-based biometrics using recurrent neural networks. In: *2017 IEEE International Conference on Acoustics, Speech and Signal Processing, ICASSP 2017, New Orleans, LA, USA, March 5–9, 2017*. IEEE (2017). p. 2062–6.

[34] Labati RD Ballester EM Piuri V Sassi R Scotti F. Deep-ecg: convolutional neural networks for ECG biometric recognition. Pattern Recognit Lett. (2019) 126:78–85. 10.1016/j.patrec.2018.03.028

[35] Ingale M Cordeiro R Thentu S Park Y Karimian N. ECG biometric authentication: a comparative analysis. IEEE Access. (2020) 8:117853–66. 10.1109/ACCESS.2020.3004464

[36] Hong P-L Hsiao J-Y Chung C-H Feng Y-M Wu S-C. ECG biometric recognition: template-free approaches based on deep learning. In: *41st Annual International Conference of the IEEE Engineering in Medicine and Biology Society, EMBC 2019, Berlin, Germany, July 23–27, 2019*. IEEE (2019). p. 2633–6. 10.1109/EMBC.2019.8856916 31946436

[37] Szegedy C Liu W Jia Y Sermanet P Reed SE Anguelov D, et al. Going deeper with convolutions. In: *IEEE Conference on Computer Vision and Pattern Recognition, CVPR 2015, Boston, MA, USA, June 7–12, 2015*. IEEE Computer Society (2015). p. 1–9.

[38] Aleidan AA Abbas Q Daadaa Y Qureshi I Perumal G Ibrahim MEA, et al. Biometric-based human identification using ensemble-based technique and ECG signals. Appl Sci. (2023) 13(16):9454. 10.3390/app13169454

[39] Agrawal V Hazratifard M Elmiligi H Gebali F. Electrocardiogram (ECG)-based user authentication using deep learning algorithms. Diagnostics. (2023) 13(3):439. 10.3390/diagnostics13030439 36766544 PMC9914224

[40] Goldberger AL Amaral LAN Glass L Hausdorff JM Ivanov PC Mark RG, et al. Physiobank, physiotoolkit, and physionet: components of a new research resource for complex physiologic signals. Circulation. (2000) 101(23):e215–20. 10851218 10.1161/01.cir.101.23.e215

[41] Moody GB Mark RG. The impact of the mit-bih arrhythmia database. IEEE Eng Med Biol Mag. (2001) 20(3):45–50. 10.1109/51.932724 11446209

[42] Fariha MAZ Ikeura R Hayakawa S Tsutsumi S. Analysis of pan-tompkins algorithm performance with noisy ecg signals. In: *Journal of Physics: Conference Series*. IOP Publishing (2020). Vol. 1532. p. 012022.

[43] Srivastva R Singh A Singh YN. Plexnet: a fast and robust ECG biometric system for human recognition. Inf Sci. (2021) 558:208–28. 10.1016/j.ins.2021.01.001

[44] Zulfiqar M Butt MFU Rama A Shafi I. Abnormality detection in cardiac signals using pseudo Wigner-Ville distribution with pre-trained convolutional neural network. In: Wysocki TA, Wysocki BJ, editors. *13th International Conference on Signal Processing and Communication Systems, ICSPCS 2019, Gold Coast, Australia, December 16–18, 2019*. IEEE (2019). p. 1–5.

[45] Chen JY Li BZ. The short-time wigner-ville distribution. Signal Process. (2024) 219:109402. 10.1016/j.sigpro.2024.109402

[46] Xin H-C Li B-Z. On a new wigner-ville distribution associated with linear canonical transform. EURASIP J Adv Signal Process. (2021) 2021(1):56. 10.1186/s13634-021-00753-3

[47] Zhang Y Zhao Z Guo C Huang J Xu K. ECG biometrics method based on convolutional neural network and transfer learning. In: *2019 International Conference on Machine Learning and Cybernetics, ICMLC 2019, Kobe, Japan, July 7–10, 2019*. IEEE (2019). p. 1–7.

[48] Akdeniz F Kayikçioglu I Kaya I Kayikçioglu T. Using Wigner-Ville distribution in ECG arrhythmia detection for telemedicine applications. In: *39th International Conference on Telecommunications and Signal Processing, TSP 2016, Vienna, Austria, June 27–29, 2016*. IEEE (2016). p. 409–12.

[49] Desai RR Gaikwad CJ Sangle SB. ECG signal classification using smoothed pseudo wigner-ville distribution. In: *2024 Second International Conference on Data Science and Information System (ICDSIS)*. IEEE (2024). p. 1–6.

[50] Zhang Q Zhou D Zeng X. Heartid: a multiresolution convolutional neural network for ecg-based biometric human identification in smart health applications. IEEE Access. (2017) 5:11805–16. 10.1109/ACCESS.2017.2707460

[51] Mai V Khalil I Meli CL. ECG biometric using multilayer perceptron and radial basis function neural networks. In: *33rd Annual International Conference of the IEEE Engineering in Medicine and Biology Society, EMBC 2011, Boston, MA, USA, August 30 – Sept. 3, 2011*. IEEE (2011). p. 2745–8. 10.1109/IEMBS.2011.6090752 22254909

[52] Lynn HM Pan SB Kim P. A deep bidirectional GRU network model for biometric electrocardiogram classification based on recurrent neural networks. IEEE Access. (2019) 7:145395–405. 10.1109/ACCESS.2019.2939947

[53] G Yang RL Wang K Huang Y Yuan F Yin Y. Robust ECG biometrics using GNMF and sparse representation. Pattern Recognit Lett. (2020) 129:70–6. 10.1016/j.patrec.2019.11.005

[54] Belo D Bento N Silva H Fred A Gamboa H. ECG biometrics using deep learning and relative score threshold classification. Sensors. (2020) 20(15):4078. 10.3390/s20154078 32707861 PMC7435887

[55] Wang K Yang G Huang Y Yin Y. Multi-scale differential feature for ECG biometrics with collective matrix factorization. Pattern Recognit. (2020) 102:107211. 10.1016/j.patcog.2020.107211

[56] Wang X Cai W Wang M. A novel approach for biometric recognition based on ECG feature vectors. Biomed Signal Process Control. (2023) 86(Part A):104922. 10.1016/j.bspc.2023.104922

[57] Fatimah B Singh P Singhal A Pachori RB. Biometric identification from ECG signals using fourier decomposition and machine learning. IEEE Trans Instrum Meas. (2022) 71:1–9. 10.1109/TIM.2022.3199260

[58] El Boujnouni I Zili H Tali A Tali T Laaziz Y. A wavelet-based capsule neural network for ECG biometric identification. Biomed Signal Process Control. (2022) 76:103692. 10.1016/j.bspc.2022.103692

[59] Meltzer D Luengo D. Efficient clustering-based electrocardiographic biometric identification. Expert Syst Appl. (2023) 219:119609. 10.1016/j.eswa.2023.119609

[60] Li J. Open medical big data and open consent and their impact on privacy. In: Karypis G, Zhang J, editors. *2017 IEEE International Congress on Big Data, BigData Congress 2017, Honolulu, HI, USA, June 25–30, 2017*. IEEE Computer Society (2017). p. 511–4.

[61] Mocydlarz-Adamcewicz M Fundowicz M Galas-Świdurska D Skrobaa A Malicki J. Respecting patients’ privacy rights and medical data safety at a radiation oncology department during remote consultations and surveillance. Int J Radiat Oncol Biol Phys. (2024) 120(2):e562–3. 10.1016/j.ijrobp.2024.07.1246

